# The impact of stress hyperglycemia ratio on short-term and long-term outcomes for acute basilar artery occlusion underwent endovascular treatment

**DOI:** 10.1186/s12883-024-03527-0

**Published:** 2024-01-12

**Authors:** Zhouzhou Peng, Yan Tian, Jinrong Hu, Jie Yang, Linyu Li, Jiacheng Huang, Weilin Kong, Changwei Guo, Xiang Liu, Dahong Yang, Chengsong Yue, Nizhen Yu, Fengli Li, Wenjie Zi, Jiaxing Song, Qingwu Yang

**Affiliations:** Department of Neurology, Xinqiao Hospital and The Second Affiliated Hospital, Army Medical University (Third Military Medical University), No.183 Xinqiao Main Street, Shapingba District, Chongqing, 400037 China

**Keywords:** Acute basilar artery occlusion, Endovascular treatment, Stress hyperglycemia ratio, Stroke

## Abstract

**Background:**

Stress hyperglycemia ratio (SHR) reflects a true acute hyperglycemic state during acute basilar artery occlusion (ABAO). We aimed to investigate the association between SHR and short-term and long-term outcomes in patients with ABAO receiving endovascular treatment (EVT).

**Methods:**

We selected patients treated with EVT from the BASILAR study, a nationwide prospective registry. A total 250 patients with documented glucose and glycated hemoglobin (HbA1C) values at admission were included. SHR was calculated as the ratio of glucose/HbA1C. All 250 patients completed 90 days of follow-up and 234 patients (93.6%) completed 1 year of follow-up. The primary outcome was the favorable outcome defined as modified Rankin Scale (mRS) score ≤ 3 at 90 days. Safety outcomes included mortality at 90 days and 1 year, and intracranial hemorrhage.

**Results:**

Among the 250 patients included, patients with higher tertiles of SHR were associated with decreased odds of a favorable functional outcome at 90 days (adjusted OR, 0.26; 95% CI, 0.12–0.56;* P* = 0.001 and adjusted OR, 0.37; 95% CI, 0.18–0.80;* P* = 0.01; respectively) and 1 year (adjusted OR, 0.34; 95% CI, 0.16–0.73; *P* = 0.006 and adjusted OR, 0.38; 95% CI, 0.18–0.82; *P* = 0.01; respectively) after adjusting for confounding covariates. The mortality was comparable across tertiles of SHR groups at 90 days and 1 year.

**Conclusions:**

Our study showed that SHR was associated with a decreased probability of favorable functional outcome both at 90 days and 1 year after EVT in patients with ABAO. The relationship was more pronounced in non-diabetes patients.

**Trial registration:**

Clinical Trial Registry Identifier: ChiCTR1800014759 (November 12, 2013).

**Supplementary Information:**

The online version contains supplementary material available at 10.1186/s12883-024-03527-0.

## Background

Acute basilar artery occlusion (ABAO) is a rare but catastrophic type of stroke that accounts for 1% of all ischemic strokes, and approximately 68% of patients die or survive with severe disability [1]. As two recent randomized controlled trials have confirmed endovascular therapy (EVT) as an effective strategy for ABAO [2, 3], it is critical to identify potential risk factors of poor outcomes and develop targeted therapeutics.

A large proportion of acute stroke patients may develop hyperglycemia, even without a history of diabetes mellitus [4]. This relatively transient increase in glucose, referred to as stress hyperglycemia, would further exacerbate brain damage and worsen the disease prognosis, which has previously been reported to be associated with poor clinical outcomes at 90 days after stroke [5]. It has also been demonstrated in animal experiments that lowering glucose by insulin further reduces brain injury after stroke, suggesting that stress hyperglycemia may be a potentially modifiable risk factor [6]. Since glycosylated hemoglobin (HbA1C) reflects background glucose status over the past 8–12 weeks, previous study showed that stress hyperglycemia ratio (SHR), measured by glucose/HbA1C, maybe a better biomarker to quantify stroke severity than absolute hyperglycemia [7]. However, most studies focused on the association of SHR with short-term outcomes after stroke, the long-term functional recovery remains unclear.

In this study, we aimed to investigate the impact of SHR on short-term and long-term outcomes in patients with ABAO treated with EVT, using the data from the Endovascular Treatment for Acute Basilar Artery Occlusion study (BASILAR).

## Methods

### Study design and participants

This cohort study used data from the BASILAR study, a nationwide prospective registry including 47 Chinese comprehensive stroke centers and an individual database of 829 consecutive adult patients with ABAO confirmed by radiology from January 2014 to May 2019. The BASILAR study was registered in the Chinese Clinical Trial Registry (http://www.chictr.org.cn; ChiCTR1800014759). The details of this registration study protocol and inclusion and exclusion criteria have been previously published [1].The study followed the Strengthening the Reporting of Observational Studies in Epidemiology (STROBE) reporting guideline for cohort studies.Of the 647 patients (78.0%) in the EVT group, 397 of 647 patients (61.4%) without admission glucose or HbA1C were excluded. Finally, 250 of 647 patients (38.6%) were included in this subgroup (SFigure 1).

The study protocol was approved by the ethics committees of the Xinqiao Hospital, Army Medical University, in Chongqing, China, and participating centers. Besides, written informed consent was obtained from all the patients or legally authorized representatives.

### Baseline data collection and assessment of SHR

Demographic data, cardiovascular risk factors, stroke severity (assessed by the National Institutes of Health Stroke Scale [NIHSS], SFigure 4) [8], posterior circulation-Alberta Stroke Program Early scores (pc-ASPECTS), laboratory tests, radiographic and angiographic data, stroke etiology, and time metrics were collected. Cardiovascular risk factors included diabetes mellitus, hypertension, hyperlipidemia, smoking, atrial fibrillation, transient ischemic attack, and coronary heart disease. Laboratory tests results, including glucose and HbA1C levels, were obtained by reviewing the patient’s first electronic medical record after admission.

Stress hyperglycemia at the time of stroke was measured by SHR, calculated using the following formula: glucose(mmol/L)/HbA1C (%). HbAlc was used to distinguish between background hyperglycemia and normal glycemia. Patients were then divided into three groups according to the tertiles of the glucose/HbA1C ratio (T1-T3) for further statistical analysis. SHR can better quantify the extent of post-stroke hyperglycemia compared to background glucose levels.

### Study outcomes and follow-up

The primary efficacy outcome was a favorable outcome at 90 days, defined as an modified Rankin Scale (mRS) score of 0–3. Secondary efficacy outcomes included mRS score of 0–3 at 1 year follow-up and mRS score distribution both at 90 days and 1 year. Safety outcomes included incidence of technique complication events (arterial perforation, arterial dissection, distal embolization, cerebral vasospasm, and vascular rupture), intracranial hemorrhage after EVT and mortality at 90 days and 1 year. The mRS score is a 7-level categorical scale for the assessment of neurological functional disability, ranging from 0 (no symptoms) to 6 (death), with functional improvement defined as a decrease in mRS score by one grade (SFigure 3) [9]. The mRS score was assessed by trained local neurologists who were blinded to the treatment-group assignments. All included 250 of 250 patients (100%) completed 90 days of follow-up, 16 of 250 patients (6.4%) were lost at 1 year, and the remaining 234 of 250 patients (93.6%) completed 1 year of follow-up.

### Statistical analyses

For baseline characteristics and outcomes, normally distributed continuous variables were reported as the mean ± SD, while non-normally distributed continuous variables and ordinal variables were presented as median and interquartile range (IQR) and categorical variables were reported as absolute numbers and percentages. Demographic and clinical characteristics were analyzed using tertiles of SHR levels with χ2 or Fisher exact tests for categorical variables and the Kruskal Wallis or Mann–Whitney U test for continuous variables.

The impact of trichotomized SHR subgroups on binary outcomes were assessed using binary logistic regression model. In the multivariable regression analyses, we adjusted for age, baseline NIHSS score, stroke etiology and occlusion site in model 1 and further adjusted for history of diabetes mellitus and intravenous thrombolysis in model 2. We additionally adjusted for sex and successful recanalization in model 3. These three models were used to assess the independent association between SHR and outcomes after controlling for other confounding factors. We performed tests for linear trend by entering the median value of each category of SHR as a continuous variable in the models. Furthermore, an ordinal regression model was used to calculate the common odds ratio for a shift towards a 1-point improvement of mRS.

In addition, the interaction between covariates and SHR in subgroups were explored using multivariate logistic regression. We calculated SHR levels as continuous values with other variables in the model set to mean values, plotted the probabilities of good functional outcomes and mortality at 90 days and 1 year of follow-up, and presented adjusted odds ratios (ORs) and 95% CIs. We performed survival analysis to show the probability of survival in different trichotomous groups of SHR at 1 year follow-up, and the results were visualized using Kaplan–Meier curves.

Statistical analyses were performed using SPSS Statistics version 26 (IBM) and the R software (version 4.2.1; https://www.r-project.org). The level of statistical significance was considered at 2-tailed *P* < 0.05. We excluded patients with missing essential data from our analysis, so we did not impute for missing data.

## Results

### Baseline characteristics

Of 829 patients enrolled in BASILAR registry, 647 (78.0%) patients were in the EVT group. 397 of 647 patients (61.4%) had missing admission glucose or HbA1C values were therefore excluded from this subgroup analysis. In total, 250 of 647 patients (38.6%) were included in this study. The median (IQR) SHR was 1.23 (1.05–1.47). Patients were then divided into 3 groups according to the tertiles of SHR for further analysis: 83 of 250 patients (33.2%) had an SHR ≤ 1.11, 83 of 250 patients (33.2%) had an SHR value of 1.12–1.36, and 84 of 250 patients (33.6%) with SHR value more than 1.37.

Baseline characteristics of the patients according to tertiles of SHR are shown in Table 1. There were 188 men (75.2%) among all eligible patients and the median (IQR) age were 64 (57–74), 66 (55–75) and 66 (57–73) years in the lower, medium and upper group, respectively. The median (IQR) baseline NIHSS score and pc-ASPECTS were 23 (14–32) and 8 (7–9). Among all 250 enrolled patients, there were 175 (70.0%), 105 (42.0%), 98 (39.2%), 50 (20.0%), 4 (1.6%) and 36 (14.4%) patients with history of hypertension, hyperlipidemia, smoking, atrial fibrillation, transient ischemic attack and coronary heart disease, respectively. Patients with higher SHR values were more likely to have received intravenous thrombolytic therapy (7 [8.4%], 19 [22.9%] and 11 [13.1%] in the lower, medium and upper group, respectively, *P* = 0.03).

**Table 1:** Baseline characteristic of patients according to the tertiles of stress hyperglycemia ratio

Variables	All patients	T1 (≤ 1.11)	T2 (1.12–1.36)	T3 (≥ 1.37)	*P* value
	250	83	83	84	
Age (median [IQR])	65 (57–74)	64 (57- 74)	66 (55- 75)	66 (57–73)	0.90
Male, n (%)	188/250 (75.2)	63/83 (75.9)	65/83 (78.3)	60/84 (71.4)	0.58
BP on admission^a^					
SBP (median [IQR])	149.00 (131.00–167.50)	146.50 (130.00–163.00)	153.00 (139.00- 168.00)	146.00 (130.00- 169.75)	0.38
DBP (median [IQR])	84.00 (78.00- 98.50)	83.00 (75.75- 96.25)	86.00 (79.00- 101.00)	83.00 (75.25- 99.50)	0.53
Glucose (median [IQR])	7.31 (6.07–9.19)	5.72 (5.37–6.28)	7.26 (6.78–8.18)	10.11 (8.37–13.00)	< 0.001
HbA1C (median [IQR])	5.90 (5.50–6.60)	5.90 (5.50–6.60)	5.90 (5.60–6.40)	5.95 (5.50–7.18)	0.89
Diabetes-related information					
History of diabetes mellitus	63/250 (25.2)	16/83 (19.3)	17/83 (20.5)	30/84 (35.7)	0.02
Diabetes treated prior to stroke	26/63 (41.3)	8/16 (50.0)	9/17 (52.9)	9/30 (30.0)	0.22
Treatment					0.22
Insulin therapy	5/26 (19.2)	2/8 (25.0)	NA	3/9 (33.3)	
Oral hypoglycemic agent	21/26 (80.8)	6/8 (75.0)	9/9 (100.0)	6/9 (66.7)	
Baseline NIHSS score (median [IQR])	23.00 (14.00–32.00)	21.00 (11.00–29.00)	24.00 (14.00–32.00)	24.50 (15.25–32.00)	0.29
Baseline pc-ASPECTS (median [IQR])^b^	8.00 (7.00–9.00)	8.00 (7.00–10.00)	8.00 (7.00–10.00)	8.00 (7.00–9.00)	0.13
PC-CS score (median [IQR])	5.00 (3.00–6.00)	5.00 (4.00–6.00)	5.00 (3.00- 6.00)	5.00 (3.00–6.00)	0.56
BATMAN score (median [IQR])	5.00 (3.00–6.00)	4.00 (3.00- 6.00)	4.00 (3.00- 6.00)	5.00 (3.00- 6.00)	0.73
Medical history (%)					
Hypertension	175/250 (70.0)	59/83 (71.1)	57/83 (68.7)	59/84 (70.2)	0.94
Hyperlipidemia	105/250 (42.0)	30/83 (36.1)	40/83 (48.2)	35/84 (41.7)	0.29
Smoking	98/250 (39.2)	36/83 (43.4)	34/83 (41.0)	28/84 (33.3)	0.38
Atrial fibrillation	50/250 (20.0)	16/83 (19.3)	16/83 (19.3)	18/84 (21.4)	0.92
Transient ischemic attack	4/250 (1.6)	1/83 (1.2)	1/83 (1.2)	2/84 (2.4)	> 0.99
Coronary heart disease	36/250 (14.4)	10/83 (12.0)	14/83 (16.9)	12/84 (14.3)	0.68
Stroke Etiology (%)					0.60
LAA	167/250 (66.8)	56/83 (67.5)	60/83 (72.3)	51/84 (60.7)	
CE	66/250 (26.4)	21/83 (25.3)	18/83 (21.7)	27/84 (32.1)	
Other causes	17/250 (6.8)	6/83 (7.2)	5/83 (6.0)	6/84 (7.1)	
Occlusion Site (%)					0.76
Distal BA	77/250 (30.8)	26/83 (31.3)	24/83 (28.9)	27/84 (32.1)	
Middle BA	74/250 (29.6)	28/83 (33.7)	25/83 (30.1)	21/84 (25.0)	
Proximal BA	52/250 (20.8)	18/83 (21.7)	16/83 (19.3)	18/84 (21.4)	
VA-V4	47/250 (18.8)	11/83 (13.3)	18/83 (21.7)	18/84 (21.4)	
IVT (%)	37/250 (14.8)	7/83 (8.4)	19/83 (22.9)	11/84 (13.1)	0.03
Recanalization (%)	204/250 (81.6)	68/83 (81.9)	63/83 (75.9)	73/84 (86.9)	0.19
OTP (median [IQR], min)^c^	315.00 (219.50–495.50)	336.00 (220.00–475.00)	313.50 (213.00–505.25)	312.00 (222.25–495.75)	> 0.99
OTR (median [IQR], min)^d^	433.00 (320.00–631.00)	430.00 (324.00–625.00)	459.50 (332.25–665.00)	429.50 (298.00–636.25)	0.57

*Abbreviations: SBP* Systolic blood pressure, *DBP* Diastolic blood pressure, *NIHSS* National Institutes of Health Stroke Scale, *pc-ASPECTS* Posterior circulation Alberta Stroke Program Early Computed Tomography Score, *pc-CS score* Posterior circulation collateral system score, *BATMAN score* Basilar artery on tomography angiography, *LAA* Large artery atherosclerosis, *CE* Cardio-embolism, *BA* Basilar artery, *VA-V4* Vertebral artery V4 segment, *IVT* Intravenous thrombolysis, *OTP* Onset to puncture, *OTR* Onset to recanalization, *IQR* Interquartile range

^a^Data were missing for 1 patient in the T1 group

^b^Data were missing for 1 patient in the T2 group and 1 patient in the T3 group

^c^Data were missing for 1 patient in the T2 group

^d^Data were missing for 1 patient in the T2 group

The upper SHR group patients had higher blood glucose levels at admission than those in the lower and medium group (median glucose, 5.72, 7.26 and 10.11 mmol/L in patients with the lower, medium and upper tertiles of SHR, respectively, *P* < 0.001). Patients in the higher and medium groups had significantly higher rates of diabetes mellitus (30 [35.7]% vs 17 [20.5%] vs 16 [19.3%], respectively, *P* = 0.02). 26 of 63 diabetes mellitus patients (41.3%) received treatment prior to the stroke,with 5 of 26 treatment patients (19.2%) received insulin therapy and the remaining 21 of 26 patients (80.8%) received oral hypoglycemic agent treatment. Other detailed patient characteristics are summarized in Table 1. Besides, the incidence of technical complication events by tertiles of stress hyperglycemia ratio were presented in STable 2.

### Impact of SHR on short-term clinical outcomes

**Fig. 1:**
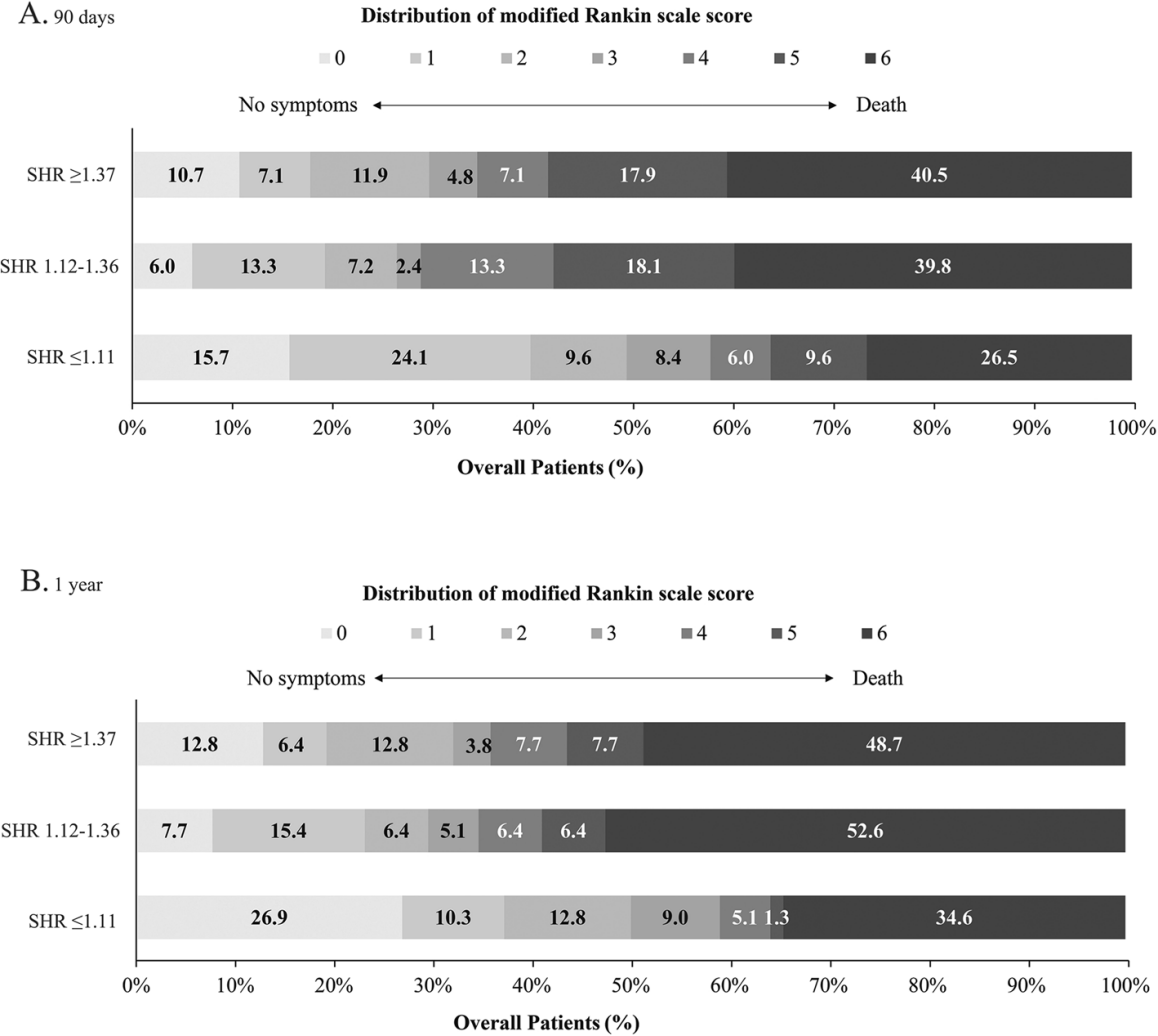
Modified Rankin Scale scores at 90 days and 1 year. The distributions of the modified Rankin Scale (mRS) score for favorable clinical outcome and mortality at 90 days (**A**) and 1 year (**B**) among patients after EVT were presented according to the trichotomized SHR. SHR indicates stress hyperglycemia ratio

**Fig. 2:**
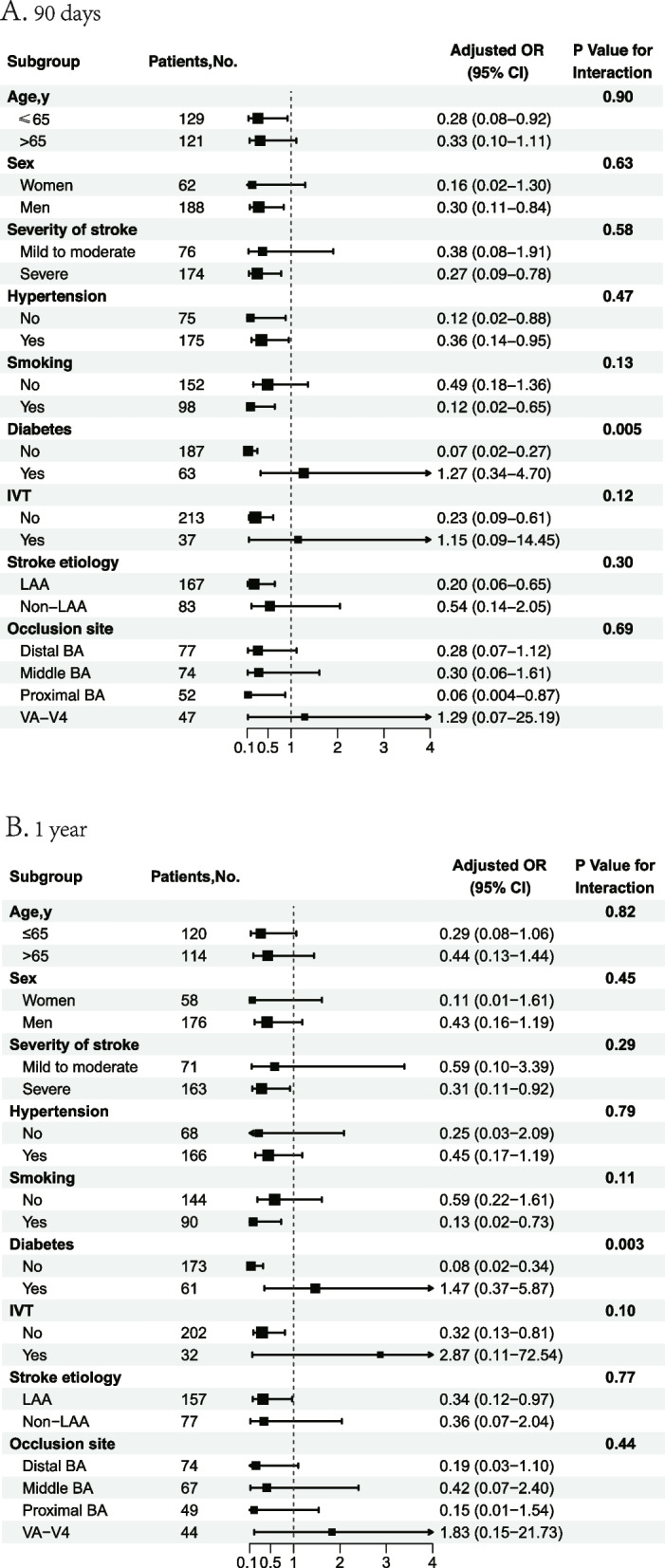
Subgroup analyses of clinical outcomes. The forest plot shows the differences in odds ratios (ORs) for favorable outcome at 90 days (**A**) and 1 year (**B**) in subgroups. Adjusted variables include age, sex, severity of stroke, hypertension, smoking, diabetes mellitus, intravenous thrombolysis, stroke etiology and occlusion site. LAA, large artery atherosclerosis; BA, basilar artery; VA-V4, vertebral artery V4 segment, IVT, intravenous thrombolysis

Table 2 shows the associations between different groups of SHR levels and clinical outcomes. Obviously, the high value of SHR was associated with poor outcome (*P* for trend = 0.004). 101 of 250 patients (40.4%) had a favorable functional outcome during 90-day follow-up. Compared with patients in the lowest tertile, patients in the highest tertile of SHR were approximately ≈0.37-fold more likely to have a favorable outcome at 3 months after fully adjusting for confounding covariates (adjusted OR, 0.37; 95% CI, 0.18–0.80; *P* = 0.01). Similar results were observed in the analysis of ordinal mRS (Table 2). In the sensitivity analysis further adjusted for diabetes mellitus and intravenous thrombolysis in Model 2 and additionally adjusted for sex and successful recanalization in Model 3, similar results were observed at each endpoint event (Table 2, Model 2 and Model 3). Significant association was not observed between tertiles of SHR and mortality at 90 days. The relationship between SHR as a binary and continuous variable and outcomes was shown in STable 3. Intracranial hemorrhage after EVT according to the tertiles of SHR was shown in STable 1. Figure 1A demonstrates the distribution of 90-day mRS scores across tertiles of SHR in the three populations. We found diabetes might alter the correlation between SHR and the primary outcome (*P* for interaction = 0.005). SHR was independently associated with unfavorable outcome at 90 days (OR = 0.07, 95% CI [0.02–0.27]) in non-diabetes.There was no other significant heterogeneity in the results of the subgroup analysis for any of the subgroups (Fig. 2A). Furthermore, as SHR values increased, the probability of estimating a favorable functional outcome decreased, while the probability of estimating death increased (SFigure 2).

**Table 2:** Clinical outcomes at 90 days and 1 year

SHR levels	Frequencies	Crude Model		Model 1^a^		Model 2^b^		Model 3^c^	
		Unadjusted OR (95% CI)	*P* value	Adjusted OR (95% CI)	*P* value	Adjusted OR (95% CI)	*P* value	Adjusted OR (95% CI)	*P* value
**90 days**									
**mRS score of 0–3, No. (%)**									
T1 (≤ 1.11)	48 (57.8)	Reference		Reference		Reference		Reference	
T2 (1.12–1.36)	24 (28.9)	0.30 (0.16–0.57)	< 0.001	0.26 (0.12–0.56)	0.001	0.25 (0.11–0.55)	0.001	0.25 (0.11–0.56)	0.001
T3 (≥ 1.37)	29 (34.5)	0.38 (0.21–0.72)	0.003	0.37 (0.18–0.80)	0.01	0.37 (0.17–0.80)	0.01	0.33 (0.15–0.74)	0.007
* P* for trend			0.004		0.01		0.01		0.01
mRS score at 90 days, median (IQR)^*^									
T1 (≤ 1.11)	3 (1–6)	Reference		Reference		Reference		Reference	
T2 (1.12–1.36)	5 (2–6)	0.42 (0.24–0.72)	0.002	0.45 (0.26–0.79)	0.006	0.44 (0.25–0.79)	0.006	0.45 (0.25–0.82)	0.009
T3 (≥ 1.37)	5 (2–6)	0.44 (0.25–0.76)	0.003	0.54 (0.31–0.96)	0.04	0.57 (0.32–1.01)	0.054	0.45 (0.25–0.82)	0.009
*P* for trend			0.005		0.046		0.07		0.01
**Mortality, No. (%)**									
T1 (≤ 1.11)	22 (26.5)	Reference		Reference		Reference		Reference	
T2 (1.12–1.36)	33 (39.8)	1.83 (0.95–3.53)	0.07	1.68 (0.82–3.44)	0.15	1.81 (0.87–3.75)	0.11	1.89 (0.83–4.32)	0.13
T3 (≥ 1.37)	34 (40.5)	1.89 (0.98–3.63)	0.06	1.59 (0.78–3.25)	0.20	1.54 (0.74–3.17)	0.25	2.14 (0.95–4.83)	0.07
*P* for trend			0.07		0.24		0.30		0.08
**1 year**									
**mRS score of 0–3, No. (%)**									
T1 (≤ 1.11)	46 (59.0)	Reference		Reference		Reference		Reference	
T2 (1.12–1.36)	27 (34.6)	0.37 (0.19–0.71)	0.003	0.34 (0.16–0.73)	0.006	0.36 (0.16–0.77)	0.009	0.37 (0.17–0.85)	0.02
T3 (≥ 1.37)	28 (35.9)	0.39 (0.20–0.74)	0.004	0.38 (0.18–0.82)	0.01	0.41 (0.19–0.88)	0.02	0.37 (0.16–0.82)	0.02
*P* for trend			0.006		0.02		0.03		0.02
**mRS score at 1 year, median (IQR)**^†^									
T1 (≤ 1.11)	3 (0–6)	Reference		Reference		Reference		Reference	
T2 (1.12–1.36)	6 (2–6)	0.41 (0.23–0.73)	0.003	0.44 (0.24–0.81)	0.009	0.45 (0.24–0.84)	0.01	0.49 (0.26–0.93)	0.03
T3 (≥ 1.37)	5 (2–6)	0.46 (0.26–0.82)	0.008	0.60 (0.33–1.10)	0.10	0.63 (0.34–1.17)	0.14	0.53 (0.28–1.00)	0.05
*P* for trend			0.01		0.12		0.17		0.06
**Mortality, No. (%)**									
T1 (≤ 1.11)	27 (34.6)	Reference		Reference		Reference		Reference	
T2 (1.12–1.36)	41 (52.6)	2.09 (1.10–3.99)	0.03	1.95 (0.97–3.93)	0.06	1.84 (0.90–3.75)	0.09	1.75 (0.80–3.81)	0.16
T3 (≥ 1.37)	38 (48.7)	1.79 (0.94–3.42)	0.08	1.52 (0.76–3.05)	0.24	1.42 (0.70–2.88)	0.34	1.73 (0.80–3.73)	0.16
*P* for trend			0.10		0.29		0.39		0.19

*Abbreviations: mRS* Modified Rankin Scale, *SHR* Stress hyperglycemia ratio, *OR* Odds ratio, *CI* Confidence interval

^*^P value for odds propotional assumption was 0.28 for mRS score at 90 days

^†^*P* value for odds propotional assumption was 0.20 for mRS score at 1 year

^a^Model 1 adjusted for age, baseline NIHSS, stroke etiology and occlusion site

^b^Model 2 adjusted for covariates from Model 1and further adjusted for history of diabetes mellitus and intravenous thrombolysis status

^c^Model 3 adjusted for covariates from Model 2 and further adjusted for sex and successful recanalization

#### Impact of SHR on long-term clinical outcomes

**Fig. 3:**
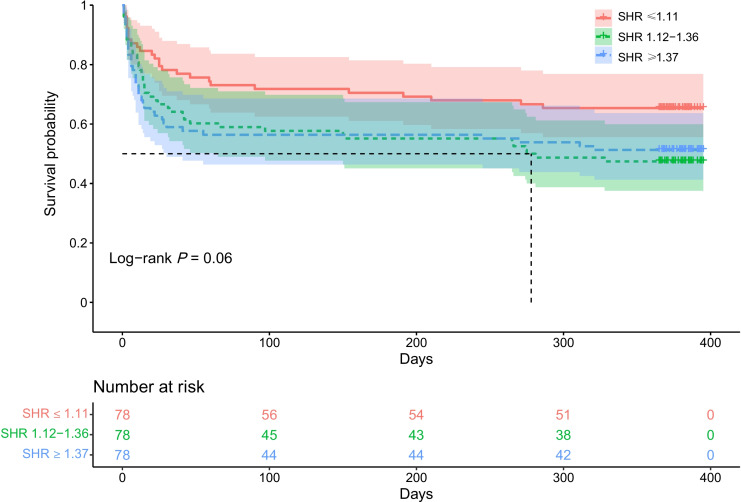
Survival probability within 1 year. Kaplan–Meier curves show the time to survival probability according to the trichotomized stress hyperglycemia ratio (SHR)

Consistent with 90-day clinical outcomes, there was a significant association between SHR levels and clinical outcomes at 1 year follow-up (*P* for trend = 0.006) (Fig. 1B). SHR levels in medium and high group were less likely to achieve a favorable functional outcome (adjusted OR, 0.34; 95% CI, 0.16–0.73; *P* = 0.006 and adjusted OR, 0.38; 95% CI, 0.18–0.82; *P* = 0.01) compared with low SHR value (Table 2). After further adjustment in Model 2 and Model 3, this association remained significant (adjusted OR, 0.36; 95% CI, 0.16–0.77; *P* = 0.009 and adjusted OR, 0.41; 95% CI, 0.19–0.88; *P* = 0.02, model 2; adjusted OR, 0.37; 95% CI, 0.17–0.85; *P* = 0.02 and adjusted OR, 0.37; 95% CI, 0.16–0.82; *P* = 0.02,model 3). Statistical significance between SHR levels and mortality at 1 year follow-up was not found in our analysis (Table 2, *P* for trend = 0.10). Similarly, Kaplan–Meier curves demonstrated that no statistically significant difference in the risk of mortality across tertiles of SHR groups within 1 year of follow-up (log-rank *P* = 0.06, Fig. 3). Subgroup analysis was shown in Fig. 2B and SHR was associated with outcome at 1 year after stroke in non-diabetic AIS (OR = 0.08, 95% CI [0.02–0.34]).

## Discussion

This study aimed to analyze the association between SHR and short-term and long-term outcomes after EVT for ABAO. In the present study, we found that the SHR value was associated with decreased probabilities of favorable functional outcome with EVT both at 90 days and 1 year. This relationship was more pronounced in non-diabetic patients.

Stress hyperglycemia is common during critical illness, such as stroke or myocardial infarction, and manifests as a sharp increase in plasma glucose due to body stress response, which would accelerate disease progression and worsen its clinical functional outcome [10–12]. This association between poor glycemic control and poor clinical outcome is particularly pronounced in patients with persistent hyperglycemia during the disease period, and with no previously known history of diabetes [13, 14]. The relationship between stress hyperglycemia and poor prognosis of clinical outcomes was firstly recognized by Melamed et al. in the 1970s [15]. Since then, many studies have successively shown that this relationship existed independently of other predictors [16, 17]. A previous systematic review has reported that 8–63% of non-diabetic ischemic stroke patients and 39–83% of diabetic ischemic stroke patients present with stress hyperglycemia on admission during the process of stroke [4]. This poorly controlled blood glucose condition may lead to aggravated cerebral damage. Furthermore, previous study also suggested that when persistent stress hyperglycemia occurs in non-diabetic patients, their neurological functional outcomes were significantly worse, with increased probability of mortality and hemorrhagic transformation [14]. However, a prospective analysis showed that the association between hyperglycemia and 3-month mortality was not significant in those who had history of poor glucose-controlled diabetes [18]. This suggests that when a history of diabetes mellitus is considered, it may alter this strongly association. Additionally, different occlusion sites and severity of stroke have an increased risk of poor outcome [19]. Therefore, in our study, occlusion site and stroke severity were adjusted, and the history of diabetes mellitus and intravenous thrombolysis were further adjusted in an additional model. Previous studies have mostly focused on the probability of short-term adverse events in stress hyperglycemia and stroke [20–22]. However, few studies have focused on the long-term adverse effects of stress hyperglycemia after a catastrophic stroke, such as ABAO. Our study further added the evidence that stress hyperglycemia was associated with decreased odds of both favorable short-term and long-term outcomes independent of other risk factors mentioned above.

Several explanations may account for the observed association between stress hyperglycemia and poor short-term and long-term outcomes after stroke. Firstly, stress hyperglycemia may be a marker reflecting the extent of ischemic damage in stroke patients [4]. Hyperglycemia in patients with critical illness represents a “stress response” to an acute event, accompanied by a massive release of neurohormonal hormones, as well as inflammatory and vasoconstrictive factors [7]. This may subsequently aggravate the severity of stroke and lead to poor outcomes. Secondly, some researchers have considered this acute elevation in glucose levels after stroke as part of a hypermetabolic syndrome, which included insulin resistance and a marked negative nitrogen balance observed both in patients and experimental animals after brain tissue injury [15, 23]. Poor long-term glycemic control in diabetic patients and further elevation of blood glucose at the time of stroke exacerbate the metabolic disorder. In patients without a diagnosis of diabetes, additionally, several studies have attributed this post-stroke admission hyperglycemia to the presence of an underlying or previously unrecognized abnormality in glucose metabolism [13]. Patients with abnormal or undiagnosed diabetes mellitus have a higher risk of developing vascular disease than those with good glycemic control. They may suffer more ischemic damage at the time of infarction due to extensive underlying cerebrovascular lesions. Thirdly, stress hyperglycemia might be accompanied by an oxidative stress generation that may give rise to the generation of an endothelial apoptosis and dysfunction, which can further lead to chronic microvascular damage in the brain [24–26]. Glucose toxicity and pathophysiological changes caused by chronic hyperglycemia in diabetic patients also exacerbate cerebral microvascular damage [27]. In addition, hyperglycemia, even in the non-diabetic range, is associated with endothelial dysfunction [28], which accounts for another possible mechanism for cerebrovascular disease in these patients. Therefore, this chronic vascular injury may be one of the potential factors contributing to its adverse long-term outcome.

Based on the growing body of evidence that has established that stress hyperglycemia is associated with poor functional outcome after stroke [16, 17, 29, 30], it is critical to identify this urgent condition and implement interventions as early as possible after the onset of the stroke. Given that blood glucose levels are influenced by many factors, it is not sufficient to define stress hyperglycemia by using a single indicator. In addition, patients’ background glucose status needs to be taken into account as well. HbA1c reflects mean glycemia over the past 8–12 weeks. As a recognized marker of elevated glucose levels, it can monitor vascular damage in diabetic patients [31]. Therefore, we used SHR, defined as glucose/HbA1C, to quantitatively measure stress hyperglycemia. Previous studies showed that this relative indicator was a better biomarker of severity in stroke patients than absolute hyperglycemia [7]. Our study presents a practical and convenient method to accurately identify stress hyperglycemia in the acute phase of stroke, providing a new strategy for the early implementation of glycemic management to improve the short-term and long-term clinical prognosis of patients.

Our study has some limitations. Firstly, the selection bias may exist inevitably, which is due to the inherent limitations of registration studies. Secondly, only Chinese patients were enrolled in the trial, which limits the generalizability of the findings to Western populations with different patterns of ischemic stroke. Thirdly, as patients were screened from multiple hospitals, the machines in different hospitals used to measure biochemical indicators are slightly different, and there may be a slight difference in the values of admission glucose and HbA1C. Fourth, we were missing data on blood glucose and HbA1C for some patients at the time of admission, as well as missing the inclusion of BMI metrics, which would have allowed us to better explore the association of SHR and clinical outcomes.

## Conclusions

Our study demonstrated that SHR (calculated as glucose/HbA1C) was associated with a decreased probability of favorable functional prognosis both at 90 days and 1 year after EVT in patients with ABAO. This relationship was more pronounced in non-diabetic patients. Therefore, early recognition of this emergency stress state and rigorous glycemic management are critical to improve short-term and long-term clinical outcomes in patients with ABAO.

### Supplementary Information

**Additional file 1: STable 1.** Intracranial hemorrhage after endovascular treatment according to the tertiles of stress hyperglycemia ratio.**STable 2.** Incidence of technical complication events according to the tertiles of stress hyperglycemia ratio. **STable 3.** Association of SHR with outcomes as a binary and continuous variable. **SFigure 1.** Flowchart of patient inclusion. **SFigure 2.** Association of stress hyperglycemia ratio with probability of clinical outcomes. **SFigure 3.** The modified Rankine Scale (mRS) score. **SFigure 4.** The National Institutes of Health Stroke Scale (NIHSS).

## Data Availability

The datasets generated during and/or analyzed during the current study are available from the corresponding author on reasonable request.

## Abbreviations

ABAO: Acute basilar artery occlusion
EVT: Endovascular treatment
HbA1C: Glycosylated hemoglobin
mRS: Modified Rankin Scale
NIHSS: National Institutes of Health Stroke Scale
pc-ASPECTS: posterior-circulation Alberta Stroke Program Early CT Score
pc-CS: posterior circulation collateral system
SHR: Stress hyperglycemia ratio
